# CD81-Receptor Associations — Impact for Hepatitis C Virus Entry and Antiviral Therapies

**DOI:** 10.3390/v6020875

**Published:** 2014-02-18

**Authors:** Laetitia Zona, Rajiv G. Tawar, Mirjam B. Zeisel, Catherine Schuster, Joachim Lupberger, Thomas F. Baumert

**Affiliations:** 1 Inserm, U1110, 67000 Strasbourg, France; E-Mails: laetitia.zona@etu.unistra.fr (L.Z.); tawar@unistra.fr (R.G.T.); mirjam.zeisel@unistra.fr (M.B.Z.); fxiao@unistra.fr (F.X.); catherine.schuster@unistra.fr (C.S.); joachim.lupberger@unistra.fr (J.L.); 2 University of Strasbourg, 67000 Strasbourg, France; 3 Pôle Hépato-digestif, Hôpitaux Universitaires de Strasbourg, 67000 Strasbourg, France

**Keywords:** antivirals, claudin-1, kinases, liver, transplantation

## Abstract

Tetraspanins are integral transmembrane proteins organized in microdomains displaying specific and direct interactions with other tetraspanins and molecular partners. Among them, CD81 has been implicated in a variety of physiological and pathological processes. CD81 also plays a crucial role in pathogen entry into host cells, including hepatitis C virus (HCV) entry into hepatocytes. HCV is a major cause of liver cirrhosis and hepatocellular carcinoma. HCV entry into hepatocytes is a complex process that requires the coordinated interaction of viral and host factors for the initiation of infection, including CD81, scavenger receptor BI, claudin-1, occludin, membrane-bound host cell kinases, Niemann-Pick C1 Like 1, Harvey rat sarcoma viral oncogene homolog (HRas), CD63 and transferrin receptor 1. Furthermore, recent data in HCV model systems have demonstrated that targeting critical components of tetraspanins and associated cell membrane proteins open new avenues to prevent and treat viral infection.

## 1. Introduction

### 1.1. The Tetraspanin Family of Proteins

Tetraspanins are part of a family of transmembrane proteins with significant sequence homology that has been conserved during evolution. In mammals, there are thirty two tetraspanins and only a minority has been extensively studied. The tetraspanin family is composed of type IV glycoproteins, discovered in the late 1980s with the subsequent cloning of CD81 as a protein of 26 kDa [1]. Tetraspanins are relatively small proteins (200–300 amino acids) composed of a small extracellular loop (SEL), a large extracellular loop (LEL), four transmembrane domains and intracellular N- and C−terminal domains [2,3]. Tetraspanins are characterized by the presence of four cysteines and a CCG motif in the LEL. The N-terminal domain forms an alpha helix containing positively charged residues important for protein interactions, as well as palmitoylation sites causing anchorage of the protein to the inner leaflet of the plasma membrane. The C-terminal domain also contains palmitoylation sites on intracellular cysteine residues [4], which are required for tetraspanin association with cholesterol complexes [5]. Palmitoylated tetraspanins are important for the assembly of the tetraspanin web by linking tetraspanins and their associated proteins in cholesterol rich regions of the plasma membrane and by their association with proteins of the cytoskeleton and signaling molecules [6,7]. 

While all cells, except sperm cells, express tetraspanins, the expression level of the individual proteins in different tissues is variable [3]. Some tetraspanins may be considered to be cell-specific while others are characterized by a very broad expression, without being ubiquitous. For example, the tetraspanin CD81 is expressed on hepatocytes, epithelial cells, fibroblasts, endothelial cells and on most of the blood cells, excluding erythrocytes, platelets and neutrophils. Tetraspanins form large complexes with other membrane proteins and given the heterogeneity in the composition as well as the dynamic nature of these complexes, tetraspanins are implicated in various biological processes, such as adhesion, migration, proliferation, signal transduction, intracellular trafficking and differentiation.

### 1.2. Interaction of CD81 with Other Host Factors

Several tetraspanins interact with other tetraspanins and partner transmembrane proteins like integrins, molecules of the immunoglobulin superfamily, cellular enzymes, signaling molecules and precursors of growth factors [5,8,9,10,11,12,13]. These interactions are direct and highly specific. Specialized regions on the surface of the cell membrane where tetraspanin interactions take place are termed as “tetraspanin-enriched microdomains” (TEMs) [3]. The composition of TEMs is cell- and tissue-specific. Thus, in each cell type, these networks consist of different tetraspanin-associated partners, which define their function. TEMs are highly regulated structures governed by cholesterol and lipid composition, by physiological stage of the cell and by palmitoylation of putative sites in juxtamembrane domains [6,7,14].

CD81 is a key tetraspanin protein expressed in numerous cell types, either alone or as a part of TEM. It is involved in myriad of physiological functions through association with other tetraspanins and membrane proteins. Among the known interaction partners of CD81 are integrin α4β1 [15] and members of the immunoglobulin family EWI-F and EWI-2 [16,17] that link CD81 to the actin cytoskeleton, thereby regulating cell motility and polarity [18,19]. On the surface of a B-cell, CD81 participates in forming CD19-signaling complex, which in conjunction with the B-cell antigen receptor (BCR) lowers the activation threshold of BCR, leading to antibody production in response to antigenic stimulation. While CD81 does not affect B-cell and T-cell development in CD81-knock-out mice, it regulates lymphocyte proliferation through multiple ways. Thus, CD81 deficiency results in enhanced antibody response to type II T-independent antigens but impaired antibody response to T-dependent antigens in CD81-null mice [20,21,22]. In line with this, a case study reported absence of CD19 expression in a patient with normal CD19 gene but possessing a rare homozygous CD81 gene defect as a cause of profound hypogammaglobulinemia [23]. 

Interestingly, CD81 is also able to activate intracellular signaling pathways, such as the mitogen−activated protein kinase (MAPK) pathway. Indeed, CD81 recruits Src homology 2 domain containing transforming protein (Shc) to the plasma membrane via its phosphotyrosine-binding (PTB) domain and induces activation of extracellular signal-regulated kinases (Erk) leading to tumor cell proliferation [24]. In addition, activated protein kinase C (PKC) migrates to the plasma membrane and associates with tetraspanins CD9, CD53, CD81, CD82 and CD151 [25]. PKC is required for integrin−mediated cell adhesion, but the formation of tetraspanin-PKC complexes is not integrin−dependent. Tetraspanins function rather as linker molecules that recruit PKC to a close proximity of integrin β1 (ITGB1) by associating ITGB1 to the extracellular domain of tetraspanins and PKC to their cytoplasmic domain [11].

## 2. Co-Receptor Association(s) and HCV Entry

### 2.1. CD81-HCV Interactions

Tetraspanins are not only essential for cell biology; they are also involved in various steps of pathogen infection including parasites, bacteria and viruses. The tetraspanin CD81 plays a role in *Plasmodium* sporozoite infection [26,27] and in *Listeria monocytogenes* entry [28]. Regarding viral pathogens, it is established that CD81 is an entry factor for hepatitis C virus (HCV) [29,30,31]. Contrarily, CD81 has been shown to negatively regulate human immunodeficiency virus-1 (HIV-1) infection by modulating envelope-mediated membrane fusion [32].

HCV infection is a leading cause of liver cirrhosis and hepatocellular carcinoma (HCC) world-wide [33,34,35]. HCV is a small enveloped virus possessing a single-stranded positive sense RNA genome. It belongs to the hepacivirus genus within the *Flaviviridae* family. *De novo* infection of hepatocytes by HCV is facilitated by two mechanisms, namely cell-free and cell-cell transmission [36,37]. Both modes of transmission rely on the viral envelope glycoproteins E1 and E2 and several host cell entry factors including CD81, scavenger receptor class B type 1 (SR-BI), claudin-1 (CLDN1), occludin (OCLN), epidermal growth factor receptor (EGFR) and its signal transducer Harvey rat sarcoma viral oncogene homolog (HRas) [37,38,39,40,41,42]. Within the past years, the molecular mechanisms of cell-free entry and the subsequent steps of the viral life cycle have been intensively characterized. Upon interaction with specific cellular receptors via its envelope glycoproteins, HCV particles are endocytosed. In the endocytic vesicle, low pH triggers fusion of the viral and the host membranes releasing the ~9.6 Kb viral genome into the cytoplasm of the newly infected cell [43]. The highly conserved un-translated regions (UTR) at the 5' and 3' ends mediate replication of the viral genome and translation of viral proteins. Internal ribosomal entry site (IRES)-dependent translation of HCV genome results in a ~3,010 amino acid polyprotein that is cleaved by host and viral proteases to yield 10 mature viral proteins consisting of three structural proteins (the core and glycoproteins E1 and E2), six non-structural proteins (NS2, NS3, NS4A, NS4B, NS5A and NS5B) and a small integral transmembrane protein (p7). Viral replication and assembly occurs at the endoplasmic reticulum (ER) membrane and in close association with lipid droplets (LDs) [44,45,46,47]. Assembly and release of HCV particles appear to be closely linked with very low density lipoprotein (VLDL) secretory pathway [48,49,50]. The released viral particles can then infect neighboring hepatocytes via cell-free infection. Of note, these viral particles are sensitive to neutralizing antibodies targeting the viral envelope glycoproteins. In addition, assembled viral particles can also be directly transmitted from an infected cell to an adjacent cell in a process that is resistant to most of the neutralizing antibodies uncovered to date, but the underlying molecular mechanisms have not been fully characterized. While cell-free infection plays an important role during initiation of infection, cell−cell transmission is thought to play a major role during maintenance of infection and viral dissemination.

The tetraspanin CD81was the first reported host factor interacting with a soluble form of the HCV glycoprotein E2 [29]. It was subsequently shown that CD81 is required for HCV infection of hepatocytes. Indeed, HCV entry and infectivity is inhibited in a pan-genotypic manner by CD81−specific antibodies [38,43,51,52,53,54,55], by a soluble recombinant form of the CD81 LEL [43,56], and by silencing CD81 expression [31]. In contrast, CD81 expression confers susceptibility to HCV infection in hepatoma cell lines lacking CD81, such as HepG2 cells [31,57,58]. Furthermore, CD81 expression levels have been shown to affect the efficiency of HCV entry [59,60]. Interestingly, a recent study demonstrated modulation of HCV RNA replication depending on CD81 expression [61]. These results suggest multiple and diverse roles of CD81 in the HCV life-cycle. 

**Figure 1:**
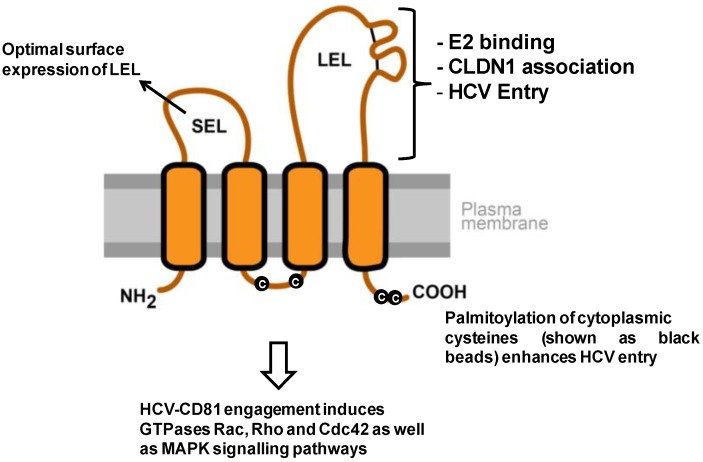
Model of CD81 topology and its relevance for HCV entry. The depicted CD81 regions comprise the cytoplasmic N- and C-terminus, a small extracellular loop (SEL), a large extracellular loop (LEL), four transmembrane domains and a cytoplasmic loop. The two disulfide bonds in the LEL are shown as black lines. The LEL mediates multiple functions that are important for HCV entry. It binds HCV envelope glycoprotein E2 (L162, K171, I181, I182, N184, F186, D196) and mediates viral entry (K171, I181, I182, F186 [64,66,71,72]). Additionally, CD81 LEL is implicated in CLDN1 association (T149, E152, T153 [73]). The SEL plays an indirect role in HCV entry by facilitating optimal expression of LEL on the cell surface by proper translocation of CD81 during its synthesis [65,74]. The post-translational palmitoylation of the cysteines (shown as black beads) and the transmembrane domains of CD81 have also been shown to enhance HCV entry [66,74]. Finally, CD81 engagement by HCV/soluble E2/CD81-specific antibodies has been shown to activate GTPases Rac, Rho and Cdc42, as well as the MAPK signaling pathways [75,76].

Various studies identified regions and residues of CD81 involved in the interaction with E2 and the viral particle (Figure 1). Indeed, E2 interacts with the LEL of CD81. E2-CD81 interaction is specific, since E2 does not bind other tetraspanins such as CD9 or CD151 [29,30,31,62,63,64]. Moreover, whereas CD81 LEL plays a direct role in HCV infection by mediating E2 binding, CD81 SEL plays an indirect role by regulating the optimal cell surface expression of LEL [65]. Several other regions of CD81, such as the C-terminal region, transmembrane residues and post-translational modification (e.g., palmitoylation of cysteines in the juxtamembrane domain) have been shown to be important for HCV entry via indirect mechanisms e.g., by mediating oligomerization of CD81, by facilitating interaction with other proteins and by cholesterol partitioning [66]. It is worth noting that residues in transmembrane domains and/or cysteine-mediated palmitoylation seem to exert only moderate inhibitory effects on HCV entry. This indicates that CD81 LEL is the key determinant of viral entry and that additional regions of CD81 only enhance viral entry.

CD81 expression on the cell surface is regulated by the membrane lipid composition. Sphingolipids associate with cholesterol to form lipid rafts and thus are important for plasma membrane organization. It has been shown that enrichment of ceramide in the plasma membrane induces internalization of CD81, thereby inhibiting HCV entry [67]. The cholesterol content of the plasma membrane is also important for HCV entry. It has been demonstrated that depletion of cholesterol—that is required for maintaining membrane fluidity—from cellular membranes inhibits HCV infection. This correlates with decreased amounts of CD81 at the cell surface since CD81 physically interacts with cholesterol [5,68]. Furthermore, it has been shown that the dynamic nature of CD81 and its lateral diffusion is dependent on cell polarization and correlate with HCV infection [69]. In addition, recent data suggest a role of CD81 trafficking in the HCV entry process [70]. Indeed, CD81 engagement with HCV or a CD81−specific antibody promotes clathrin-dependent internalization of CD81. Interestingly, the CD81−specific antibody also appears to neutralize HCV after its internalization, suggesting that intracellular CD81 plays a role in HCV infection [70].

### 2.2. The Tetraspanin Complex Formation during HCV Entry and Downstream Signaling Pathways

HCV relies on multiple host factors to gain entry into hepatocytes (reviewed in [77]). This includes two molecules of the tetraspanin superfamily, namely CD81 and CD63. Interestingly, both tetraspanins have been shown to interact with the viral envelope glycoprotein E2, and may thus directly interact with HCV during the viral entry process [29,78]. While CD81 does not bind any known endogenous ligand nor possess an internalization motif, binding of HCV glycoprotein E2 or CD81-specific antibodies to CD81 has been shown to activate GTPases Rac, Rho and Cdc42 as well as the MAPK signaling pathways [75,76]. These cellular events could regulate HCV interactions with its co−receptors and establish viral entry into target cell. In addition, CD63 is involved in clathrin−dependent endocytosis and vesicle trafficking to lysosomes, suggesting that CD63 may facilitate HCV uptake. Furthermore, while CD81 and CD63 have been involved in TEM formation [79], it is not yet known whether they interact with each other in hepatocytes. 

Besides the potential direct effect in HCV envelope glycoprotein binding and subsequent downstream events described above, CD81 has also been shown to contribute to HCV entry by forming a co-receptor complex through its interaction with other proteins. A major breakthrough was the identification of the CD81-CLDN1 co-receptor complex [80,81]. Noteworthy, while there is no physiological role for this complex known to date, the association of CD81 to CLDN1 is a mandatory step of the HCV entry process. CLDN1 is an integral transmembrane protein of 25 kDa with membrane topology similar to CD81 [82]. However, it is not classified as a classical tetraspanin because of the lack of four cysteines and a CCG motif in the extracellular loop two, one of the defining features of tetraspanins. It has been demonstrated that CLDN1 is an essential host cell factor for HCV entry as cells lacking CLDN1 are resistant to HCV [83]. However, in contrast to CD81, CLDN1 is not seen as a classical HCV receptor as it does not directly bind to soluble HCV glycoprotein E2, a key viral protein involved in HCV entry. Neither CD81 nor CLDN1 appear to bind infectious viral particles [83,84], probably due to the masking of the viral envelope by host-derived lipoproteins, however, a recent study reported that E1E2 complexes are able to interact with CLDN1 [85]. These data suggest that CLDN1, like CD81, may contribute to HCV envelope glycoprotein binding, but that both proteins recognize distinct parts of the viral envelope. While predominantly expressed at the tight junction (TJ), CLDN1 also localizes on the basolateral membrane of hepatocytes. Noteworthy, this pool of CLDN1 outside of the TJ co-localizes with CD81 and allows the formation of the CD81−CLDN1 co-receptor complex that is essential for HCV entry [80,81,84]. Interestingly, CLDN1 co-localizes not only with CD81 at the plasma membrane, but also with SR-BI, another important HCV entry factor, suggesting that these HCV entry factors may be part of a larger membrane complex important for viral entry [42,86,87].

**Figure 2:**
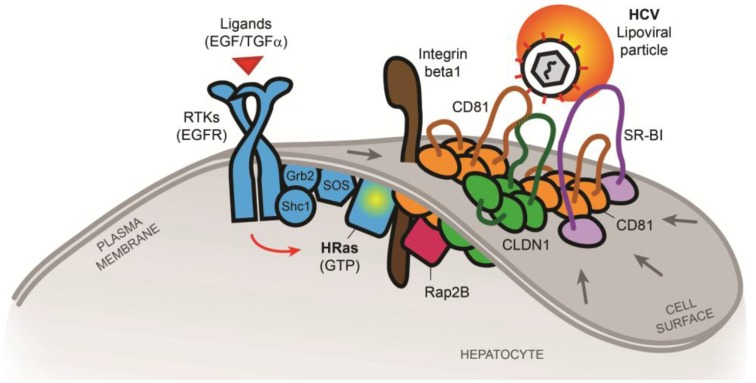
Model of tetraspanin co-receptor formation(s) and HCV entry according to [42]. RTK signaling mediated by e.g., EGFR is relevant for viral entry including HCV [41]. HRas, recruited and activated by EGFR via the scaffolding proteins Shc1 and Grb2, act as a key host signaling transducer for viral entry. HRas signaling modulates lateral membrane diffusion of CD81 and promotes CD81-CLDN1 co-receptor complex formation that is essential for HCV entry. Moreover, HRas is associated with TEMs containing host receptors CD81, CLDN1, SR-BI and the previously unknown HCV entry factors integrin beta 1 (ITGB1) and Rap2B. Viruses may thus exploit HRas signaling for cellular entry by compartmentalization of entry factors and receptor trafficking. This highlights a new mechanism to regulate CD81-dependent pathogen invasion of the liver [42].

A genome-wide host kinase RNAi screen demonstrated an important regulatory role of kinases for HCV entry and infection [41]. Several kinases have been implicated in CLDN1 cellular localization, relocation and CD81-CLDN1 co-receptor complex formation including protein kinase A (PKA) [88] and receptor tyrosine kinases (RTKs) [41]. Inhibition of EGFR kinase activity by erlotinib or silencing EGFR expression reduces CD81 association to CLDN1 at the cell surface [41]. This highlights a role of EGFR signaling in the formation of the CD81-CLDN1 co-receptor complex and subsequent HCV entry. While EGFR is known to activate many downstream signals in various cell types, it has been demonstrated that EGFR predominantly activates MAPK signaling in hepatocytes [42]. Indeed, HRas GTPase, a molecular switch for the activation of the MAPK pathway, was identified as a cellular transducer of RTK signals required for HCV entry [42]. A differential proteomic approach allowed to identify HRas as well as CLDN1, SR-BI, integrin beta 1 (ITGB1) and Rap2B as specifically CD81 TEM-associated proteins [42]. Given that all these host factors play a role in HCV entry, these data indicate the existence of a functional membrane network of proteins involved in viral entry [42]. As HRas signaling has been demonstrated to modulate lateral membrane diffusion of CD81 which allows assembly of the tetraspanin receptor complex subsequently mediating HCV entry [42], HRas appears at the crossroad of interplay between EGFR signaling and the CD81 receptor complex (Figure 2). Taken together, these findings suggest that HCV may manipulate RTK signaling to promote its propagation. Indeed, it has been shown that virus engagement to CD81 activates phosphatidylinositol-3-kinase (PI3K)/Akt pathway [89] and EGFR that could contribute to virus internalization [90]. This indicates that HCV actively influences the composition of CD81 TEMs via signaling events in order to promote its own entry into target cells. As CD81 has also been shown to be important for influenza entry and HRas has been shown to play a role in influenza [91] and measles virus [42] entry, perturbation of TEMs may represent a novel concept for the development of antiviral strategies that may be effective against several pathogens using the same machinery.

## 3. Tetraspanins as Antiviral Targets

RNA viruses including HCV, influenza virus and retroviruses exhibit profound genetic diversity resulting from error prone replication of viral genome. This enables the viral population to rapidly evolve and adapt to changes in the host environment. As a consequence, most RNA viruses quickly develop resistance to antiviral treatments and thus present a major challenge in vaccine design and antiviral drug development. In contrast, targeting host components critical for the virus life-cycle increases the genetic barrier of resistance rendering this approach an attractive alternative or complementary strategy for the development of novel antiviral drugs [92]. HCV entry factors associated within TEMs therefore represent potential targets for therapeutic intervention (Table 1).

**Table 1:** Host-targeting agents interfering with TEM functions required for HCV entry.

Compounds	Target	Stage of Development	Reference(s)
Anti-CD81 mAbs	CD81	Cell culture model	[70,93,94]
		Animal model	[95]
Imidazole-based small molecules	CD81	Cell culture model	[96]
Anti-CLDN1 mAbs	CLDN1	Cell culture model	[97]
Anti-CLDN1 peptides	CLDN1	Cell culture model	[98]
Erlotinib	EGFR	Cell culture and animal model	[41]
		Phase I/IIa	ClinicalTrials.gov: NCT01835938
Tipifarnib	Ras	Cell culture model	[42]
Anti-ITGB1 mAbs	ITGB1	Cell culture model	[42]

### 3.1. Targeting CD81 and CLDN1 to Inhibit HCV Infection

CD81 and CLDN1 are key host factors used by HCV to gain entry into hepatocytes. Antibodies targeting CD81 or CLDN1 have been shown to potently inhibit HCV entry [93,95,97,99]. Several CD81−specific antibodies previously developed exert their inhibitory effect by interfering with E2−CD81 binding [30,93]. Among these, the monoclonal antibody (mAb) JS-81 (BD Biosciences, Le Pont-de-Claix, France) has been widely studied *in vitro*. Prophylactic treatment with JS-81 mAb potently prevents HCV infection in the human-liver chimeric uPA-SCID mouse model; however, it has no effect on viraemia when administered post-infection [95]. Nevertheless, the prophylactic application of CD81-specific antibody may be exploited in liver transplantation to overcome re-infection of the graft, which remains an important challenge. However, the toxicity issues associated with blocking CD81 need to be addressed since CD81-specific antibodies were shown to elevate transaminase levels and cause syncytia formation in the human portion of the liver of chimeric uPA-SCID mice [100]. Potentially, the toxicity observed can be overcome by targeting a defined aspect of CD81 function required for HCV entry without interfering with its physiological role e.g., by using small molecule inhibitors or peptides. Indeed, several imidazole-based small molecules mimicking the D-helix of CD81 have been shown to specifically block the E2-CD81 interaction [96]. However, it remains to be determined whether these molecules can inhibit *in vitro* and *in vivo* HCV infection. 

Unlike CD81, CLDN1 is highly expressed in liver and has a more restricted tissue distribution. Furthermore, it is important for cell-free and cell-cell transmission of HCV [38,83,84,87]. This property makes CLDN1 an attractive target for both prevention and treatment of HCV infection. In fact, sera from rats genetically immunized with CLDN1 potently inhibit HCV entry by disrupting the CD81-CLDN1 co-receptor complex [84]. Subsequently, six CLDN1-specific mAbs possessing anti−HCV activity were isolated from an immunized rat [97]. Interestingly, these antibodies did not alter TJ integrity in polarized HepG2 cells nor did they lead to toxicity in primary human hepatocytes. Independently, another laboratory reported CLDN1-specific antibodies able to inhibit HCV entry. These antibodies were isolated from a functional minimalist phage display library using baculovirus particles displaying membrane anchored full length CLDN1 as a bait [99]. In addition to antibodies, a CLDN1-derived peptide, CL58, also inhibits HCV infection *in vitro* when used for prevention or treatment of HCV infection. CL58 was identified by screening a library of overlapping peptides covering the complete CLDN1 sequence for anti-HCV activity [98]. Interestingly, like CLDN1−specific antibodies, this peptide did not show any adverse effect on TJ integrity *in vitro*. Whether CL58 can mediate anti-HCV effect *in vivo* remains to be shown. Collectively, these data point to CLDN1 as a promising drug target for antiviral therapy.

### 3.2. Targeting Regulators of the HCV Co-Receptor Complex Formation as an Antiviral Strategy

Although not classified as tetraspanins, activators of the MAPK signaling pathway, in particular EGFR and GTPase HRas, have been shown to modulate TEM organization and thereby HCV entry by interacting with tetraspanins [42]. Erlotinib, a clinically approved EGFR inhibitor for cancer treatment, has been thoroughly studied for its anti-HCV activity [41]. *In vitro* studies demonstrate that erlotinib exerts its anti-HCV activity by preventing CD81-CLDN1 co-receptor complex formation, by interfering with HCV envelope glycoprotein-mediated fusion events and by blocking cell-cell transmission. Furthermore, administration of erlotinib to human-liver chimeric uPA-SCID mice markedly impaired HCV infection when given pre-challenge [41]. Notably, the anti-HCV effect of erlotinib was substantiated by a case report in which rapid virologic response was observed in a HCV−infected HCC-patient given erlotinib monotherapy following discontinuation of interferon alfa (IFN-α)-based treatment after liver transplantation [101]. A clinical trial investigating efficacy, safety and toxicity of erlotinib monotherapy in HCV patients chronically infected with genotype 1b, in treatment naïve patients and in non-responders/relapsers to IFN and/or ribavirin is currently underway (clinical trial reference number—NCT01835938). Interestingly, an *in vitro* study recently demonstrated that combinations of small doses of erlotinib and IFN-α potentiates the antiviral impact of IFN-α in a synergistic manner by promoting IFN-α-induced interferon response gene expression [102]. 

On the other hand, the GTPase HRas regulates the formation of tetraspanin-complexes by stabilizing CD81 molecules in the TEM on the surface of liver-derived cells [42]. Localized near the inner leaflet of the plasma membrane, HRas acts as an on-off switch controlling cell growth, differentiation and survival (reviewed in [103]). Farnesylation of HRas is important for its membrane anchorage following activation and thus for its function. Two classes of drugs targeting HRas have been developed—farnesyltransferase inhibitors (FTIs) that prevent farnesylation of HRas, and membrane dissociating agents that dislodge HRas from the membrane [104,105,106]. While tipifarnib, an FTI, was shown to exhibit profound anti-HCV activity, the effect of farnesylthiosalicylic acid (FTS) that displaces HRas from the cell membrane was less potent [42]. This indicates that activated (farnesylated) HRas mediates formation of a stable CD81-CLDN1 co-receptor complex, which is not affected by displacement of HRas from the membrane. Consequently, HRas represents another novel target for the development of anti-HCV drugs. Proof-of-concept studies in animal models using the HRas inhibitor tipifarnib, which is already in phase II clinical trials for the treatment of acute myeloid leukemia in elderly patients (NCT01364038), may also open the way for the development of antiviral HRas inhibitors.

### 3.3. Other Antiviral Targets in CD81 TEMs

CD81 TEM-associated proteins including HRas associated-GTPase Rap2B, the tetraspanin CD63 and ITGB1 have been demonstrated to be relevant for HCV entry and thus represent potential antiviral drug targets [42,78]. Inhibitors of Rap2B or CD63 have not yet been reported; however, once available, it would be interesting to assess their potential to inhibit HCV infection. Noteworthy, integrins are used as host entry receptors by many viruses [36]. This suggests that these viruses may enter host cells using a common mechanism that possibly requires CD81 TEM association. ITGB1−specific antibody has been recently shown to potently inhibit HCV entry *in vitro* [42]. Further, given that various drug candidates targeting different combinations of integrin a and β subunits are in pre-clinical development, it will be interesting to see if any of these compounds exhibit anti-HCV activity (reviewed in [107]).

## 4. Conclusions and Perspectives

TEMs play a role in membrane compartmentalization leading to coupling and/or regulating molecular machinery and signaling pathways in a tissue specific manner. The tetraspanin CD81 is not only a key HCV entry factor, but also a molecular organizer of plasma membrane microdomains that contain the molecular machinery used by HCV. Signal transduction through associated tetraspanins and partner proteins likely induce actin remodeling allowing lateral movement of CD81, which appears to be required for HCV entry. This suggests a cooperative action of HCV entry factors and molecules required for vesicle formation and trafficking, leading to compartmentalization of entry factors in TEMs. Moreover, recent studies have also highlighted a central role of CD81 TEMs and virus-induced host cell signaling for entry of HCV [42,90]. Taken together, these data support a model where CD81 complexes, activated either by the virus itself or by RTK signaling, provide a functional link between CD81 trafficking and CD81-CLDN1 association that are prerequisites of HCV entry and highlight a crucial role of TEM “platforms” in the HCV entry process. These findings suggest that CD81 TEMs are highly relevant for pathogen entry such as HCV. Additionally, as CD81 mediated TEMs are required by other viruses, they present potential targets for novel broad spectrum antivirals. 
